# The Genetic Deletion of 6q21 and *PRDM1* and Clinical Implications in Extranodal NK/T Cell Lymphoma, Nasal Type

**DOI:** 10.1155/2015/435423

**Published:** 2015-06-29

**Authors:** Li Liang, Zhang Zhang, Ying Wang, Lin Nong, Yalin Zheng, Linlin Qu, Bo Zhang, Ting Li

**Affiliations:** ^1^Department of Pathology, Peking University First Hospital, Beijing 100034, China; ^2^Department of Pathology, Peking University Health Science Center, Beijing 100191, China

## Abstract

6q21 genetic deletion has been frequently detected in extranodal NK/T cell lymphoma, nasal type (EN-NK/T-NT), and *PRDM1* is considered as candidate gene. However, direct detection of *PRDM1* deletion has not been well documented. We investigated genetic alterations of 6q21 and *PRDM1* in 43 cases of EN-NK/T-NT and cell lines by FISH. PRDM1 expression was evaluated by immunohistochemistry and Western blot. The correlation between genetic alteration and PRDM1 expression and the significance in clinic-pathologic were analyzed. Heterozygous deletion of 6q21 and/or *PRDM1* was observed in 24 of 43 cases (55.81%) of EN-NK/T-NT including 16 cases (37.21%) for 6q21 deletion and 19 cases (44.19%) for *PRDM1* deletion. Similarly, heterozygous codeletion of 6q21 and *PRDM1* was identified in NK92 and NKL cells. The heterozygous deletion of 6q21 and/or *PRDM1* was correlated with PRDM1 expression. However, genetic deletion of 6q21 and/or *PRDM1* was not correlated with clinicopathological features of EN-NK/T-NT, while PRDM1 expression showed positive effect on the outcome of patients as those as disease site, B symptom, and clinical stage. Thus, heterozygous deletion of 6q21 and/or *PRDM1* was frequently detected in EN-NK/T-NT and correlated with downregulation of PRDM1. But the prognostic role of genetic deletion needs to be further evaluated in larger cohort.

## 1. Introduction

Extranodal NK/T cell lymphoma, nasal type (EN-NK/T-NT), is featured by angiocentric and angiodestructive growth pattern characteristically correlated with progressive unrelenting ulceration and necrotic granulomatous alteration in midfacial tissues. Clinically, this lymphoma exhibits highly aggressive clinical course, poor outcome, and a predilection for East Asians and Central and South Americans [1]. Our recent investigation for 142 Northern Chinese patients with peripheral NK/T cell lymphomas revealed that EN-NK/T-NT was the most prevalent subtype (38.0%) [2]. The prognosis of EN-NK/T-NT is various, greatly depending on clinical factors, such as international prognostic index, stage, therapy modes, high proliferation rate, and primary tumour location [3, 4]. However, previous reports for the prognostic significance of pathologic factors are distinct and even controversial. Although some putative oncogenic or molecular mechanisms have been incriminated to account for its high aggressiveness and pathogenesis, further elucidation of the genetic aberrations and pathological prognostic factors of this disease is needed [5–9].

Conventional cytogenetic studies in EN-NK/T-NT have showed chromosomal imbalances including deletions of 1p, 6q, 11q, 13q, and 17p and gains of 1q, 2q, 7q, 17q, and 20q [7, 10, 11], and recent studies reported that the deletion of chromosome 6q21 is the most frequent aberration in NK cell neoplasm and candidate genes in the region including* PRDM1*,* FOXO3*,* ATG5*,* AIM1*, and* HACE1 *have been investigated [7, 12–14]. Meanwhile, the inactivation of* PRDM1* has been well documented and is consequently considered to be the putative candidate gene responsible for EN-NK/T-NT. In our recent research, loss of* PRDM1* expression has been identified in a group of EN-NK/T-NT patients [15]. Nevertheless, up to date, concrete data concerning the analysis for genetic alteration of 6q21 and its putative tumor suppressor gene* PRDM1* are still lacking.

In this study, we retrospectively investigated 70 cases of EN-NK/T-NT, carried out fluorescence in situ hybridization (FISH) test on formalin-fixed paraffin-embedded (FFPE) tumor specimens and NK/T lymphoma cell lines, clarified the deletion status of chromosome 6q21 and specific* PRDM1* gene, and evaluated the clinical significances and prognostic factors.

## 2. Materials and Methods

### 2.1. Patients and Samples

70 cases of EN-NK/T-NT were collected from archives of the Department of Pathology, Peking University First Hospital. The histological specimens of all cases were fixed in 10% buffered formalin and processed for routine paraffin-embedding. Histological sections with a thickness of 4 *μ*m were stained with hematoxylin and eosin and used for immunoperoxidase procedures. Diagnosis of EN-NK/T-NT was made on the basis of combined morphological and immunophenotypical findings (including positive CD56 and cytotoxic proteins) as well as EBV positivity as determined by in situ hybridization (ISH) with an Epstein-Barr virus-encoded small RNA (EBER-1) probe, according to WHO classification [16].

The gender ratio was 1.26 with 39 males and 31 females and ages ranging from 8 to 86 years (median 43 years). Follow-up data were available for 40 patients. The follow-up period was defined as the time from the date of initial diagnosis to the patient's death from any cause or last follow-up visit.

The study was approved by the ethics committee of Peking University First Hospital (no. 2013 [571]) and was performed according to ethics committee regulations and in accordance with the Declaration of Helsinki. The patient data were obtained from the medical record library through a double-blind process and were analysed anonymously. There was no risk of conflict of interests for the patients.

### 2.2. Cell Culture

Three NK/T-cell lymphoma cell lines, YT [17], NK92 [18], NKL [19], and the human chronic myelogenous leukemia cell line K562 were used in the present study. YT and NKL cells were obtained from Beijing Hong Bokang Biological Technology (Beijing, China). NK92 and K562 cells were purchased from the Chinese Academy of Medical Sciences (Beijing, China). YT, NKL, and K562 cells were maintained in RPMI medium 1640 (Invitrogen, Carlsbad, CA, USA) with 10% fetal bovine serum (Bio-Chrome, Germany) and were cultured in humidified 37°C incubator with 5% CO_2_. NK92 cell was cultured in Alpha Minimum Essential medium (HyClone, UT, USA) with 12.5% horse serum and 12.5% fetal bovine serum. The media of NK92 and NKL cells were supplemented with 100 U/mL human recombinant IL-2 (PeproTech, London, UK).

### 2.3. FISH

Probes of 6q21,* PRDM1 *gene, and chromosome 6 (the centromere of chromosome 6) were purchased from Empire Genomics (New York, USA). For FFPE tumor specimens, dual-color FISH was performed according to the manufacturer's instructions. Briefly, tissue sections (1.5 *μ*m thick) were deparaffinized in fresh xylene, rehydrated in 100%, 95%, 80%, and 70% ethanol, and air dried. After several washes in 2x SSC, the tissue sections were incubated in dH_2_O at 90°C for 30 min and incubated with protease K at 37°C for 1 min. The slides were then rinsed in 2x SSC for 5 min and dehydrated in absolute ethanol and allowed to air dry. The slides were added with 10 *μ*L of hybridization mixture and denaturation for 4 min at 83°C and were incubated for 48 hr in Hybridization Oven (ThermoBrite) at 37°C. After being rinsed in 2x SSC/0.1% NP-40, the slides were mounted with medium containing DAPI (0.5 *μ*g/mL) for nuclear counterstaining. The cultured cells were affixed to glass slides using ThinPrep Cytology test, and 10 *μ*L of hybridization mixture was denaturized at 73°C for 5 min and hybridized at 37°C for 24 hr. For each of slides, 200 nuclei of cells were evaluated independently by two observers and the cut-off values for each probe were determined from three FFPE tonsil specimens and were defined as the mean copy number in tonsil plus three standard deviations for FFPE specimens. The cut-off baseline levels were 27.78% for 6q21 and 33.71% for* PRDM1* gene in specimens.

### 2.4. Immunohistochemistry (IHC)

IHC staining was carried out using the DAKO EnVision detection kit (Dako, Glostrup, Denmark). Tissue sections were subjected to heat-induced antigen retrieval in EDTA buffer (pH 9.0). A panel of primary antibody including PRDM1 (clone C14A4, cell signaling, Beverly, MA, USA), granzyme B (clone 11F1, Novocastra, Newcastle upon Tyne, England), TIA-1 (clone TIA-1, Coulter, Hialeah, FL, USA), and CD56 (Clone UMAB83, Zhongshan, China) was utilized in our study.

A positive nuclear staining pattern was interpreted as representing PRDM1 immunoreactivity, while the cytoplasmic immunoreactivity for granzyme B or TIA-1 was considered as positive staining. Based on Garcia and Nie's investigations [20, 21], positive expression of PRDM1 was defined as nuclear staining in 10% or more of the tumour population and was semiquantitatively estimated as follows: negative (no positive cells or <10%), weak (10% to ≤50% positive cells), or strong (>50% to 100% positive cells). Tonsils sample was used as positive control for PRDM1 staining. For the negative control reactions, phosphate buffer saline (PBS) was used instead of the primary antibody.

### 2.5. Western Blot

Whole-cell lysates of YT, NK92, NKL, and K562 cells were separated in 10% sodium dodecyl sulfate polyacrylamide gel and were transferred to polyvinylidene difluoride (Millipore, MA, USA). After being blocked with 5% skim milk, the membrane was reacted with rabbit monoclonal antibody PRDM1 (PRDI-BF1) (1 : 1,000; Cell Signaling Technology, Beverly, MA) and *β*-actin (1 : 5,000; Roche Applied Science, Indianapolis, USA). Secondary antibodies conjugated to Horseradish Peroxidase included anti-rabbit (1 : 5,000, Zhongshan, China) and anti-mouse (1 : 5,000, Zhongshan, China). PRDM1 expression was quantified by densitometry and normalized with *β*-actin.

### 2.6. Statistical Analysis

The Spearman rank correlation coefficient test was utilized to correlate the expression of PRDM1 protein and deletion of 6q21 and/or* PRDM1 *gene. Treatment outcomes were measured by overall survival (OS) and failure-free survival (FFS). OS was calculated as the time from initial diagnosis to death from any cause or last follow-up. FFS was defined as the time from initial diagnosis to progression, relapse, or death from any cause. The estimates of FFS and OS were calculated using the Kaplan-Meier method and compared to log-rank tests. Multivariate analysis was conducted using the Cox proportional hazard model to define the independent prognostic factors for OS and FFS. Differences were considered statistically significant when the 2-sided *P* value was less than 0.05. All analyses were performed using SPSS (Statistical Package for the Social Sciences) 13.0 software (Chicago, IL).

## 3. Results

### 3.1. Immunophenotyping of EN-NK/T-NT Cases

Immunohistochemically, all 70 cases showed positive staining for CD3*ε* and cytotoxic proteins (93.84% for TIA-1, 92.06% for granzyme B, and 86.15% for both), and 58 cases (82.86%) were positive for CD56. ISH detection revealed positive staining for EBER in 63 cases (90.00%) in moderate or strong intensitity. The remaining 7 EBER-negative cases expressed CD56 and cytotoxic granule proteins (TIA-1 and/or granzyme B). All 70 cases were reviewed and diagnosed by two experienced pathologists.

### 3.2. The Heterozygous Deletion of 6q21 and* PRDM1 *in EN-NK/T-NT

To disclose the cytogenetic alteration in EN-NK/T-NT specimens, 43 FFPE specimens of EN-NK/T-NT were subjected to dual-color FISH for detection of 6q21 or* PRDM1 *gene deletion, respectively. We found that the cytogenetic alteration of 6q21 and* PRDM1* gene occurred in heterozygous deletion, while no homozygous deletion was found. As shown in Table 1, 24 of 43 EN-NK/T-NT cases (55.81%) showed the heterozygous deletion of 6q21 and/or* PRDM1*, with 16 cases (37.21%) for heterozygous deletion of 6q21 and 19 cases (44.19%) for heterozygous deletion of* PRDM1* gene. 11 EN-NK/T-NT cases presented the heterozygous codeletion of 6q21 and* PRDM1 *gene. We also depicted that 5 EN-NK/T-NT cases showed only 6q21 heterozygous deletion (indicated by pound sign) and 8 cases presented only* PRDM1* gene heterozygous deletion (indicated by asterisk) in Table 1.

**Figure 1:**
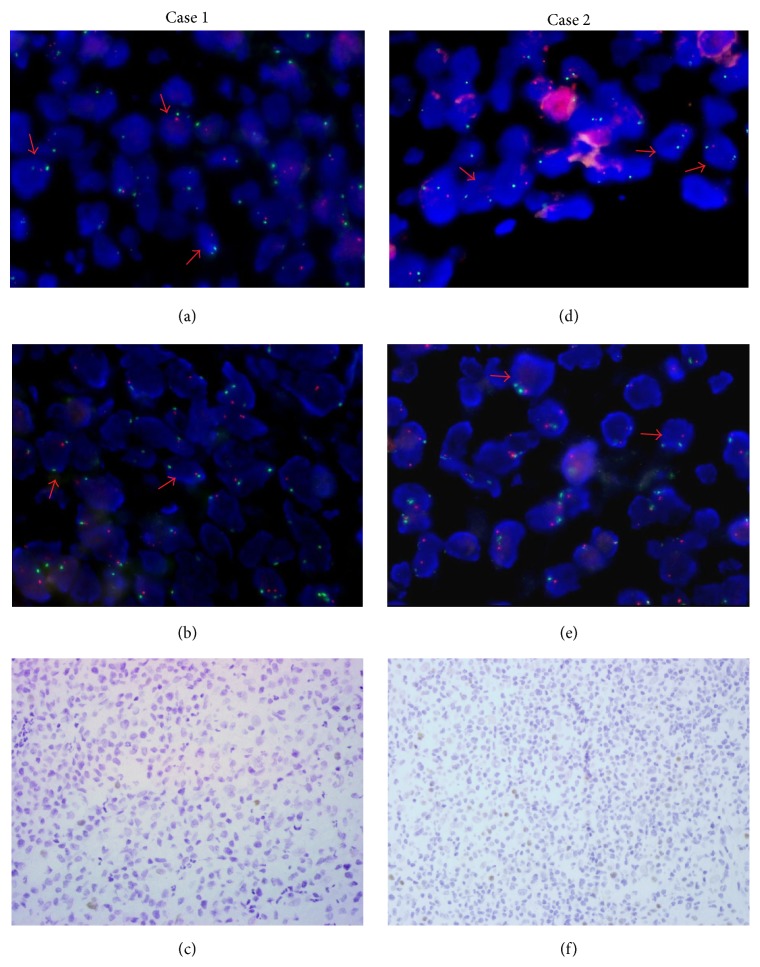
Identification of 6q21 and* PRDM1 *deletion by FISH and PRDM1 expression by immunostaining in EN-NK/T-NT cases. Dual-color FISH was performed with 6q21 probe or* PRDM1* specific probe (labelled with 5-carboxyl-xrhodamine, red) and chromosome 6 centromere specific probe (labelled with 5-fluorescein, green), respectively. Case 1, (a), (b), and (c): one representative case showed the heterozygous codeletion of 6q21 (a, two green and one red) and* PRDM1* gene (b, two green and one red) and PRDM1 negative immunostaining (c). Case 2, (d), (e), and (f): another representative case with the heterozygous codeletion of 6q21 (d, two green and one red) and* PRDM1* gene (e, two green and one red) and PRDM1 negative immunostaining (f). (a, b, d, and e at 1000x magnification; c and f at 400x magnification).

**Figure 2:**
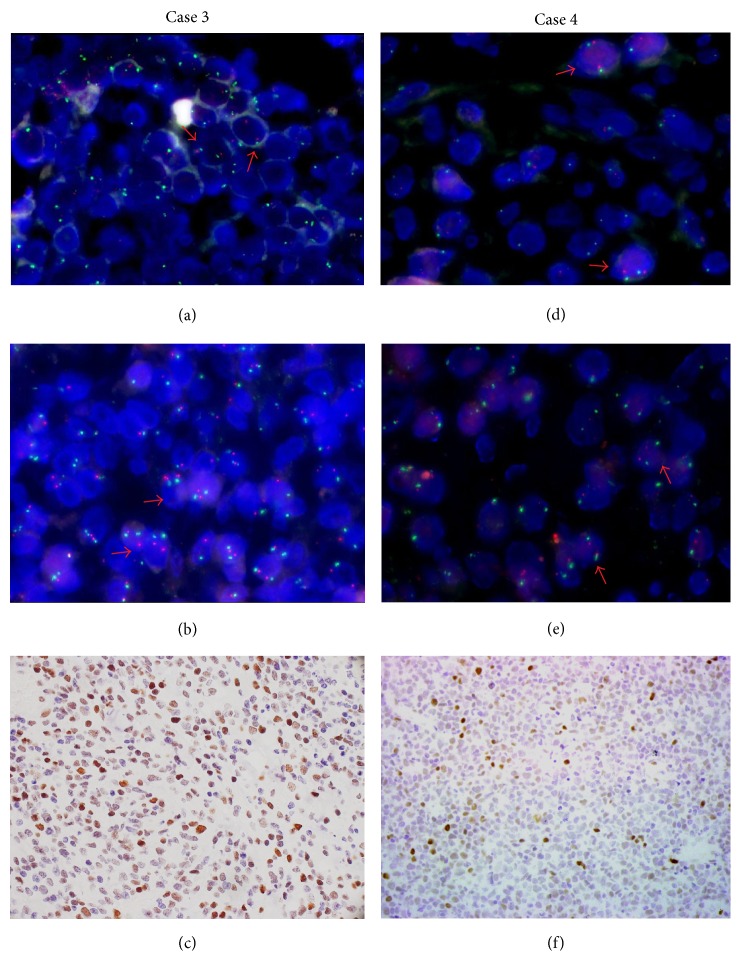
Two representative EN-NK/T-NT cases without deletion of 6q21 or* PRDM1* gene. Dual-color FISH was performed with 6q21 probe or* PRDM1* specific probe (labelled with 5-carboxyl-xrhodamine, red) and chromosome 6 centromere specific probe (labelled with 5-fluorescein, green), respectively. Case 3, (a), (b), and (c): one case without deletion of 6q21 (a, two green and two red) or* PRDM1* gene (b, two green and two red) showed PRDM1 positive immunostaining (c). Case 4, (d), (e), and (f): another representative case without deletion of 6q21 (d, two green and two red) or* PRDM1* gene (e, two green and two red) presented PRDM1 positive immunostaining (f). (a, b, d, and e at 1000x magnification; c and f at 400x magnification).

We found the significant positive correlation between 6q21 deletion and* PRDM1* deletion by Spearman rank correlation coefficient test (*P* = 0.021, *r* = 0.381). Representative FISH images of the heterozygous deletion of 6q21 and* PRDM1* gene in EN-NK/T-NT cases were displayed in Figures 1 and 2.

**Figure 3:**
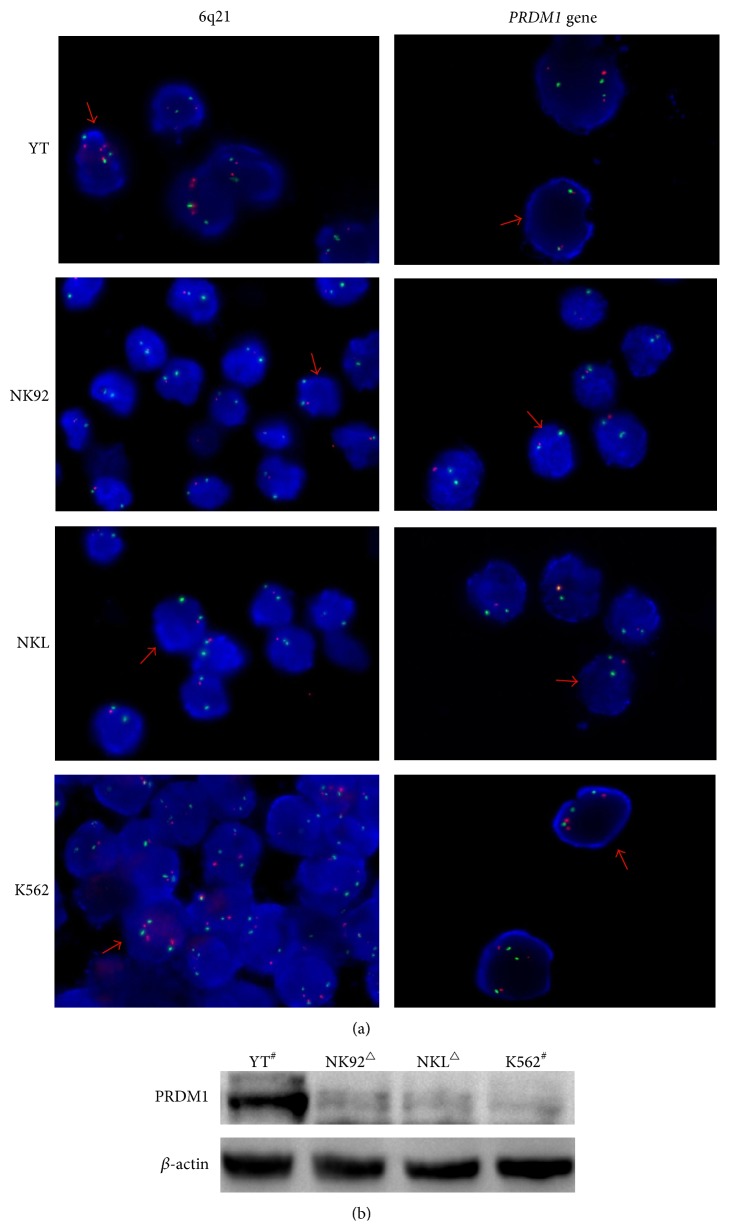
Identification of 6q21 and* PRDM1 *gene deletion by dual-color FISH and PRDM1 protein expression by Western blot in cell lines. (a) Dual-color FISH analysis was performed in NK/T cell lymphoma cell lines (YT, NK92, and NKL) and control cell line (K562, human chronic myelogenous leukemia cell) as described in Figure 1. YT cells with strong PRDM1 protein expression showed no deletion of 6q21 and* PRDM1 *gene, while NK92 and NKL cells with weak PRDM1 expression displayed the heterozygous codeletion of 6q21 and* PRDM1*. In addition, control cell line K562 lack of PRDM1 expression showed no deletion of 6q21 or* PRDM1*. All are shown at 1000x magnification. (b) Representative picture of PRDM1 protein expression by Western blot in YT, NK92, NKL, and K562 cells. ^#^No deletion of 6q21 or* PRDM1 *gene. ^△^The heterozygous codeletion of 6q21 and* PRDM1 *gene.

In addition, three NK/T lymphoma cell lines were also subjected to FISH detection. The heterozygous codeletion of 6q21 and* PRDM1* was found in NK92 and NKL cells, but no deletion was found in YT cells and control cell line K562 (Figure 3(a)).

The finding proved that 6q21 deletion in EN-NK/T-NT was generally heterozygous and was commonly involved with* PRDM1* gene.

### 3.3. The Correlation between PRDM1 Expression and the Genetic Deletion in EN-NK/T-NT

The correlation between PRDM1 expression and genetic deletion of 6q21 or* PRDM1* was analyzed in 43 EN-NK/T-NT cases. PRDM1 positive staining was observed in 4 of 24 cases (16.67%) with the deletion of 6q21 and/or* PRDM1* in comparison with 9 of 19 cases (47.38%) with no deletion (Table 2). Statistically, PRDM1 positive staining was significantly correlated with the heterogeneous deletion of 6q21 and/or* PRDM1 *(*P* = 0.030, *r* = 0.332). However, PRDM1 expression showed no correlation with the heterozygous deletion of* PRDM1 *alone (*P* = 0.254) or heterozygous codeletion of 6q21 and* PRDM1 *(*P* = 0.118), except that PRDM1 staining reversely related to heterozygous deletion of 6q21 alone in marginal statistical value (*P* = 0.053). We also found that 13 EN-NK/T-NT cases showed the heterozygous deletion of either 6q21 or* PRDM1* gene (indicated by pound sign) in Table 2, which was consistent with the data in Table 1.

Our previous investigation has demonstrated that the downregulation of PRDM1 protein was common in EN-NK/T-NT [15]. In this study, PRDM1 positive staining was observed in the nuclei of EN-NK/T-NT tumour cells and a quarter of EN-NK/T-NT cases (18/70, 25.71%) exhibited weak expression of PRDM1 (10% to ≤50% stained cells) harbouring weak to moderate intensity, and the remaining 52 cases were negative in PRDM1 staining. The findings further identified the downregulation of PRDM1 protein in EN-NK/T-NT cases.

With cultured cells, YT cells without deletion of 6q21 or* PRDM1* showed strong PRDM1 expression in Western blot, but NK92 and NKL cells with the heterozygous deletion of 6q21 and* PRDM1* showed weak expression (Figure 3(b)). However, control cell line K562 without 6q21 deletion or* PRDM1* deletion lacked PRDM1 expression.

These findings were in agreement with what we have found in EN-NK/T-NT specimens, further demonstrating that PRDM1 expression may be associated with the heterozygous deletions of 6q21 and* PRDM1 *gene.

### 3.4. The Clinical Significance of Genetic Deletion and PRDM1 Expression

The clinicopathological significance of 6q21 deletion, specific* PRDM1* gene deletion, and PRDM1 protein expression was analyzed from a follow-up cohort (40 cases) of EN-NK/T-NT cases. The median OS was 12.5 months (range, 1–120 months), and the median FFS was also 12.5 months (range, 1–87 months).

**Figure 4:**
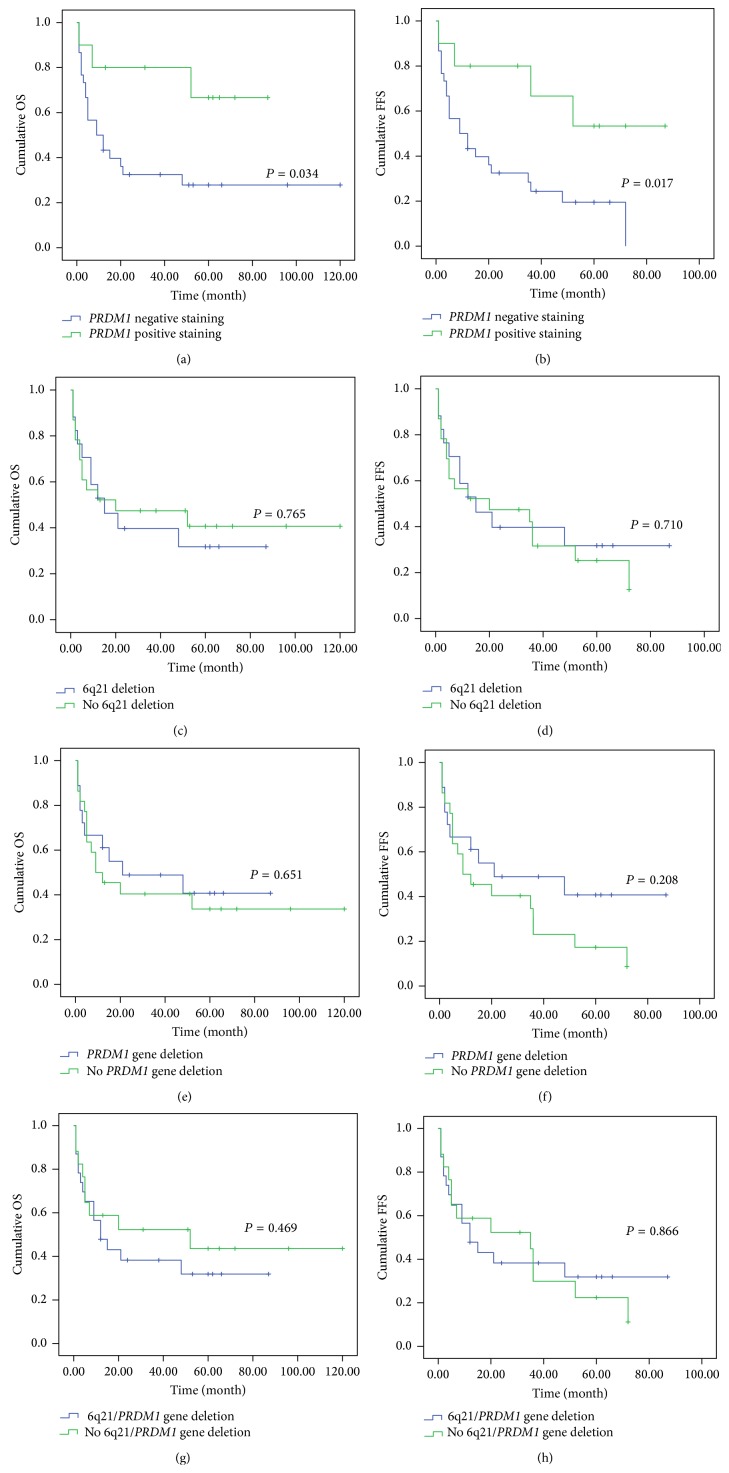
Kaplan-Meier survival analysis of PRDM1 immunostaining, 6q21 deletion, and* PRDM1* deletion in follow-up cohort of 40 EN-NK/T-NT cases. PRDM1 positive immunostaining predicted a favorable effect on overall survival (OS) (a, *P* = 0.034) and failure-free survival (FFS) (b, *P* = 0.017) of EN-NK/T-NT patients. However, the status of 6q21 (c, d) and specific* PRDM1* gene alone (e, f) showed no significant correlation with the OS and FFS as those of the deletion of 6q21 and/or specific* PRDM1* gene (g, *P* = 0.469; h, *P* = 0.866).

As shown in Table 3, 6q21 heterozygous deletion showed no significant association with sex, age, angiocentric infiltration, necrosis, site, EBER, B symptom, Ann arbor stage, and the outcome of patients (*P* > 0.05). Similar results were found between* PRDM1* heterozygous deletion and these clinical factors (Table 3). In addition, 6q21 heterozygous deletion was not significantly correlated with the OS and FFS of patients (Figure 4). Similarly, the status of specific* PRDM1* gene showed no significant correlation with the OS and FFS (Figure 4). The clinical significance of the genetic deletion with either 6q21 and/or* PRDM1 *was also analysed. No significant correlation was found among them (Table 3, Figure 4). Furthermore, the clinical significance of 11 cases with codeletion of 6q21 and PRDM1 also showed no clinical significance (data not shown).

However, the expression of PRDM1 protein in specimens was decreased significantly with progression from early Ann Arbor stage (I/II stage) to advanced Ann Arbor stage (III/IV stage) (*P* = 0.003) (Table 4). PRDM1 expression was significantly different between living and deceased patients (*P* = 0.006) (Table 4). Importantly, it was revealed that PRDM1-positive staining predicted a favorable effect on OS and FFS by Kaplan-Meier single-factor analysis and the log-rank test (Figure 4, *P* = 0.034, *P* = 0.017). Histologically, angiocentric and angioinvasive infiltration and necrosis were also commonly seen in PRDM1 negative cases, of which 27 cases (38.57%) presented angiocentric and angioinvasive infiltration, and 57 cases (81.43%) displayed necrosis. Statistically, we found that PRDM1 positive immunostaining was reversely correlated with angiocentric infiltration (*P* = 0.027, *r* = −0.265) by the Spearman rank correlation coefficient test but not with necrosis (Table 4).

**Figure 5:**
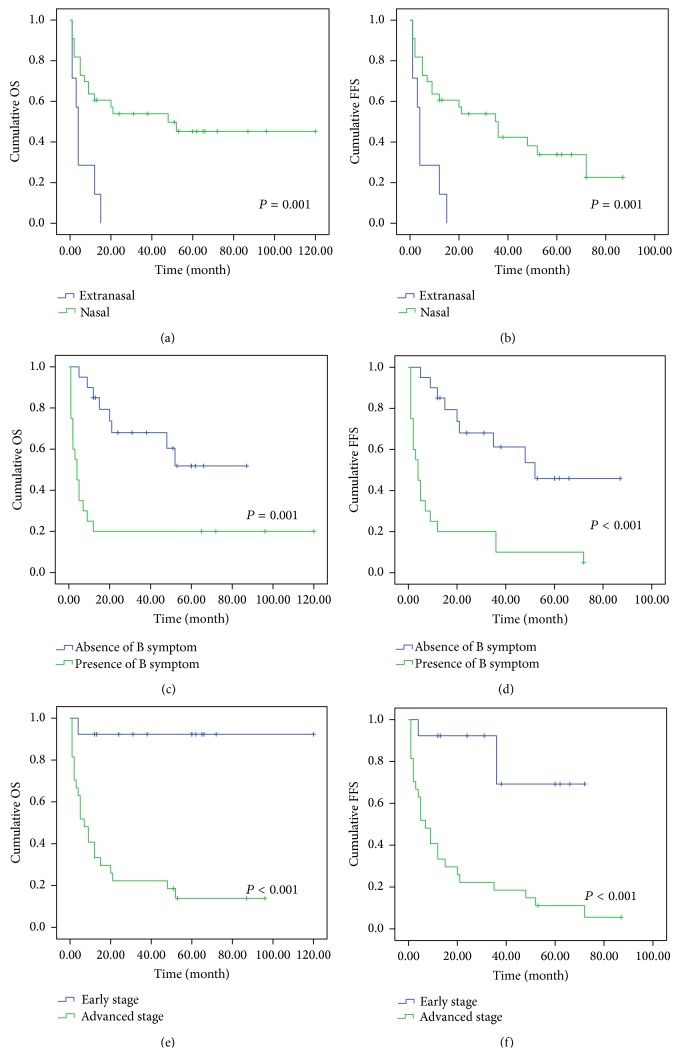
Kaplan-Meier survival analysis of disease site, B symptom, and clinical stage in 40 cases of EN-NK/T-NT. Statistically, EN-NK/T-NT patients with extranasal disease present significant correlation with poorer OS (a, *P* = 0.001) and FFS (b, *P* = 0.001). B symptom and late stage (III/IV) showed poorer OS and FFS (c, *P* = 0.001; d, *P* < 0.001; e, *P* < 0.001; f, *P* < 0.001).

We found that PRDM1 immunostaining may not be the independent predictive factor using univariate analysis; in contrary, patients with extranasal disease exhibited poorer OS and FFS than cases with nasal disease (Figure 5, *P* = 0.001, *P* = 0.001). Additionally, B symptom and late stage (III/IV) were associated with poorer OS and FFS (Figure 5, *P* = 0.001, *P* < 0.001; *P* < 0.001, *P* < 0.001). Importantly, our results demonstrated that disease site, B symptom, and clinical stage were independent predictive factors of EN-NK/T-NT patients by multivariate Cox model analysis.

## 4. Discussion

The deletion of 6q21 has been identified as the common genetic aberration in NK/T cell lymphoma (20–43%) [5, 10, 12], and the genes located in the region of 6q21, including* POPDC3*,* PREP*,* PRDM1*,* ATG5*,* AIM1*,* HACE1*, and* FOXO3*, were reasonably considered the affected candidates [7, 12, 13]. Since the downregulation of PRDM1 and FOXO3 has been clarified in cells and clinical specimens of EN-NK/T-NT, both are believed to be candidate genes involved in genomic deletion. However, these investigations were generally done by conventional cytogenetic analysis or genomic approach such as array-based comparative genomic hybridization (aCGH), and direct analysis in tumor cells has been rarely performed. In the described study, we simultaneously detected 6q21 and* PRDM1* using FISH assay in the collection of 43 cases of EN-NK/T-NT, we clarified that genetic deletion of 6q21 and/or* PRDM1 *could be found in 55.81% (24/43) of EN-NK/T-NT cases, and genetic alteration was heterozygous. In addition, we also revealed a consistence between EN-NK/T-NT specimens and* in vitro* NK/T lymphoma cell lines. Thus, FISH analysis represented a highly sensitive, time-effective, and reliable alternative for the detection of 6q21 and* PRDM1* deletion and can be utilized in routine pathologic diagnosis.

Despite that codeletion could be found in nearly half of cases, single deletion of 6q21 or* PRDM1* could be observed in some cases of EN-NK/T-NT. Similarly, Karube et al. [13] also observed that genomic deletion could vary in a large region spaning 6q15–22 at least in length of 1.5 Mb. It could be assumed that the deletion of 6q21 region should sequentially occur in development of EN-NK/T-NT. On the other hand, as we all know, besides* PRDM1*, 6q21 region also contains* POPDC3*,* PREP*,* ATG5*,* AIM1*,* HACE1,* and* FOXO3. *It is possible that 6q21 deletion should also involve above genes. Actually,* FOXO3 *gene has been identified to be heterozygous loss in NKL cell lines reported by Karube et al. [13], in which FOXO3 expression proved to be downregulated and was also regarded as a tumor suppressor gene. Karube et al. [13] suggest that FOXO3 together with PRDM1 could be considered to play more important roles as tumor suppressor genes in NK/T cell lymphoma than the other candidate genes located on 6q21. Other reports also indicated that the expression of HACE1, ATG5, and AIM1 was decreased in NK/T cell lymphoma [5, 7, 22], although the research is limited. Further studies will be needed to confirm the status of genetic alteration in this region.

As other previous reports, we also observed a discrepancy between the genetic alteration and PRDM1 staining. This could be credited to heterozygous deletion of* PRDM1*, in which the cells still have an undeleted allele. Thus, another mechanism is needed to explain the loss of PRDM1 expression. In fact, mutations of PRDM1 have been clarified in NK92 cells and some cases of EN-NK/T-NT, in which base-substitution in Exon 5 (C951A) or Exon 4 (C864A) results in nonsense mutation to generate truncated PRDM1 [7]. Meanwhile, epigenetic mechanism for inactivation of* PRDM1 *such as DNA methylation and micro-RNA silencing has also been described [12, 23]. Our previous study has revealed that the downregulation of PRDM1 protein is common and is associated with upregulated miR-223 in EN-NK/T-NT [15]. In this study, we further found that PRDM1 expression was lost in most EN-NK/T-NT cases (74.29%) and that the downregulation of PRDM1 protein may be associated with the heterozygous deletion of 6q21 or* PRDM1* gene. Thus, PRDM1 protein level could be determined by heterozygous deletion of one allele and inactivation of another allele through genomic mutations or epigenetic silencing. It is believed that more clear answer could be expected from analysis on expanded cases.

Our previous research showed that PRDM1 expression could predict significant effect on Ann Arbor stage, OS, and FFS. Notably, PRDM1 positive immunostaining was negatively correlated with angiocentric infiltration, which is the characteristic histology in EN-NK/T-NT. In contrast, the heterozygous deletion of* PRDM1* or 6q21 showed no important clinical significance and prognostic value. This is probably caused by the limited cases of FISH analysis, and further investigation in expanded cohort could give a more clear answer.

Collectively, the heterozygous deletions of 6q21 and* PRDM1* gene were identified in FFPE EN-NK/T-NT specimens and cell lines by FISH analysis and significantly correlated with downexpression of PRDM1, which could be used as a prognostic indicator in evaluation of EN-NK/T-NT as disease site, B symptom, and clinical stage.

## Figures and Tables

**Table 1:** Heterozygous deletion of 6q21 and *PRDM1 *gene in NK/T cases.

	Heterozygous deletion	No deletion	Total	*P*
	24	19	43	**0.021**
6q21	11 (5^#^)	19 (8^*∗*^)		
*PRDM1* gene	11 (8^*∗*^)	19 (5^#^)		

5^#^: 5 cases only showing 6q21 heterozygous deletion.

8^*∗*^: 8 cases only showing *PRDM1* gene heterozygous deletion.

**Table 2:** The comparison of PRDM1 expression with heterozygous deletion of 6q21 and *PRDM1*.

	Total	PRDM1 staining		*P *
		Positive	Negative	
	43	13	30	
6q21 and/or *PRDM1* HD	24	4	20	**0.030**
Negative	19	9	10	
6q21 HD	16	2	14	0.053
Negative	27	11	16	
*PRDM1* HD	19	4	15	0.254
Negative	24	9	15	
Codeletion of 6q21 and *PRDM1 *	11 (13^#^)	2 (2^#^)	9 (11^#^)	0.118
Negative	19	9	10	

^#^Cases with heterozygous deletion of either 6q21 or *PRDM1 *gene.

HD, heterozygous deletion.

**Table 3:** The clinical significance of the heterozygous deletion of 6q21 and *PRDM1* and deletion of 6q21 and *PRDM1*.

	*n*	Percent	6q21		*P*	*PRDM1 *		*P*	6q21/*PRDM1 *		*P*
			Deletion	No deletion		Deletion	No deletion		Deletion	No deletion	
Patients	43										
Male	25	58.14	9	16	0.851	13	12	0.234	15	10	0.526
Female	18	41.86	7	11		6	12		9	9	
Age (year)	43										
≤60	32	74.42	13	19	0.441	13	19	0.435	18	14	0.924
>60	11	25.58	3	8		6	5		6	5	
Angiocentric infiltration	43										
With	16	37.21	5	11	0.545	7	9	0.966	8	8	0.579
Without	27	62.79	11	16		12	15		16	11	
Necrosis	43										
With	34	79.07	13	21	0.793	16	18	0.473	20	14	0.452
Without	9	20.93	3	6		3	6		4	5	
Site	43										
Nasal	35	81.40	14	21	0.440	14	21	0.258	19	16	0.682
Extranasal	8	18.60	2	6		5	3		5	3	
EBER	43										
Positive	39	90.70	14	25	0.589	17	22	0.811	21	18	0.429
Negative	4	9.30	2	2		2	2		3	1	
B symptom	40										
With	20	50.00	5	15	0.055	6	14	0.059	9	11	0.115
Without	20	50.00	11	9		12	8		14	6	
Ann arbor Stage	43										
I/II	14	32.56	5	9	0.729	7	7	0.604	7	7	0.604
III/IV	29	67.44	12	17		12	17		17	12	
Outcome	40										
Alive	14	35.00	5	9	0.536	6	8	0.864	6	8	0.178
Dead	26	65.00	12	14		12	14		17	9	

**Table 4:** The clinical significance of PRDM1 expression.

	*n*	Percent (%)	PRDM1 expression		*P*
			Negative	Positive	
Patients	70				
Male	39	55.71	30	9	0.578
Female	31	44.29	22	9	
Age (year)	70				
≤60	53	75.71	41	12	0.472
>60	17	24.29	11	6	
Angiocentric infiltration	70				
With	27	38.57	24	3	**0.027 **
Without	43	61.43	28	15	
Necrosis	70				
With	57	81.43	44	13	0.250
Without	13	18.57	8	5	
Site	70				
Nasal	57	81.43	39	18	**0.046 **
Extranasal	13	18.57	13	0	
B symptom	40				
With	20	50.00	16	4	0.478
Without	20	50.00	14	6	
Ann arbor Stage	57				
I/II	22	38.60	10	12	**0.003 **
III/IV	35	61.40	29	6	
Outcome	40				
Alive	14	35.00	7	7	**0.006 **
Dead	26	65.00	23	3	
